# Imaging the enteric nervous system

**DOI:** 10.3389/fnana.2025.1532900

**Published:** 2025-03-12

**Authors:** Doriane Hazart, Marwa Moulzir, Brigitte Delhomme, Martin Oheim, Clément Ricard

**Affiliations:** ^1^Université Paris Cité, CNRS, Saints-Pères Paris Institute for the Neurosciences, Paris, France; ^2^Doctoral School Brain, Cognition and Behaviour – ED3C - ED 158, Paris, France

**Keywords:** 3-D imaging, histological method, whole-mount preparation technique, in-vivo imaging, clearing method, intestine

## Abstract

The enteric nervous system (ENS) has garnered increasing scientific interest due to its pivotal role in digestive processes and its involvement in various gastrointestinal and central nervous system (CNS) disorders, including Crohn’s disease, Parkinson’s disease, and autism. Despite its significance, the ENS remains relatively underexplored by neurobiologists, primarily because its structure and function are less understood compared to the CNS. This review examines both pioneering methodologies that initially revealed the intricate layered structure of the ENS and recent advancements in studying its three-dimensional (3-D) organization, both in fixed samples and at a functional level, *ex-vivo* or *in-vivo*. Traditionally, imaging the ENS relied on histological techniques involving sequential tissue sectioning, staining, and microscopic imaging of single sections. However, this method has limitations representing the full complexity of the ENS’s 3-D meshwork, which led to the development of more intact preparations, such as whole-mount preparation, as well as the use of volume imaging techniques. Advancements in 3-D imaging, particularly methods like spinning-disk confocal, 2-photon, and light-sheet microscopies, combined with tissue-clearing techniques, have revolutionized our understanding of the ENS’s fine structure. These approaches offer detailed views of its cellular architecture, including interactions among various cell types, blood vessels, and lymphatic vessels. They have also enhanced our comprehension of ENS-related pathologies, such as inflammatory bowel disease, Hirschsprung’s disease (HSCR), and the ENS’s involvement in neurodegenerative disorders like Parkinson’s (PD) and Alzheimer’s diseases (AD). More recently, 2-photon or confocal *in-vivo* imaging, combined with transgenic approaches for calcium imaging, or confocal laser endomicroscopy, have opened new avenues for functional studies of the ENS. These methods enable real-time observation of enteric neuronal and glial activity and their interactions. While routinely used in CNS studies, their application to understanding local circuits and signals in the ENS is relatively recent and presents unique challenges, such as accommodating peristaltic movements. Advancements in 3-D *in-vivo* functional imaging are expected to significantly deepen our understanding of the ENS and its roles in gastrointestinal and neurological diseases, potentially leading to improved diagnostic and therapeutic strategies.

## 1 Introduction

**Figure 1:**
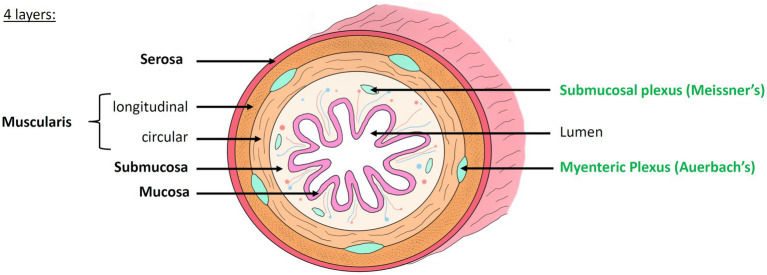
Histology of the intestinal wall and anatomy of the enteric nervous system. The intestinal wall is organized into 4 concentric layers: the mucosa, submucosa, muscularis and serosa. The mucosa, composed of many villi, is the most internal layer, in direct contact with the lumen of the digestive tract. The submucosa is a highly vascularized connective tissue. It contains the submucosal plexus (Meissner’s plexus). The muscularis is composed of two layers of smooth muscle whose orientation changes: the inner circular layer and the outer longitudinal layer. Between these two layers lies the myenteric plexus (Auerbach’s plexus). The serosa is the outermost layer. Modified from Hazart et al. (2024).

For a century, the study of the nervous system and the understanding of mental disorders and neurodegenerative diseases has been almost exclusively focusing on the central nervous system (CNS). Since the 1960s, however the scientific community has begun to no longer ignore another part of the nervous system, essential for the proper functioning of the body, and located in the digestive tract: the enteric nervous system (ENS) (Gershon, 1999; Avetisyan et al., 2015; Schneider et al., 2019; Wattchow et al., 2022; Sharkey and Mawe, 2023). The ENS, a quasi-autonomous nervous system (which communicate with the CNS but whose function is independent from it), is composed of hundreds of millions of neurons and glial cells located all along the intestinal wall. The ENS forms a concentric, layered and ganglionated meshwork (Figure 1) that controls the motility and secretions throughout the digestive tract (Furness, 2012; Wattchow et al., 2022). We distinguish two layers:

- The myenteric plexus, or Auerbach’s plexus, located between the two *muscularis* layers, which modulates the contraction of the smooth muscles and thus ensures *peristalsis* and segmentation (Auerbach, 1862)
- The submucosal plexus, or Meissner’s plexus, located in the *submucosa*, which mainly controls the secretory functions of the gastrointestinal tract (Von Kölliker, 1863; Neckel, 2023)

The ENS can act independently from the central, sympathetic and parasympathetic nervous systems, but it is also influenced by them.

Crucial for the normal functioning of digestion, nutrient uptake and immune response, the ENS is involved in numerous pathologic conditions, too. It can present molecular or structural anomalies leading to dysfunctions which have been associated with a number of gastrointestinal pathologies, including, e.g., Hirschsprung’s disease (HSCR) (Diposarosa et al., 2021; Ji et al., 2021) or chronic inflammatory bowel diseases (IBD) (Sharkey and Kroese, 2001; Jang et al., 2018; Mogilevski et al., 2019; Oligschlaeger et al., 2019), the later include *morbus Crohn* and ulcerative colitis. Perhaps more surprising, dysfunctions of the ENS are also observed in certain central pathologies with no apparent immediate link to the digestive tract, such as Alzheimer’s (AD) (Rao and Gershon, 2016; Nielsen et al., 2021) and Parkinson’s diseases (PD) (Rao and Gershon, 2016; Nielsen et al., 2021; Horsager et al., 2022; Warnecke et al., 2022), or autism spectrum disorders (Rao and Gershon, 2016; Nielsen et al., 2021). Moreover, many psychological disorders, such as anxiety, may be linked to ENS via imbalances in the intestinal microbiota (Simpson et al., 2021). Notably, the intestinal microbiota, which refers to the collection of microorganisms living in the digestive tract—including bacteria, viruses, fungi, and archaea—plays an important role in digestion, metabolism (synthesis of certain vitamins like B12 and K), immunity, and protection against pathogens.

Its balance is influenced by diet, medications (notably antibiotics), and lifestyle. Having drawn significant attention from the scientific community in recent years, it has been observed that an imbalance in the intestine microbiota could be associated with various diseases, such as digestive disorders (IBD), metabolic diseases (diabetes, obesity), and even neurological disorders via the gut-brain axis. The interactions of ENS neural functions and the microbial are a vast field of research, out of focus in this review (see, however for review Cryan et al., 2019; Heiss and Olofsson, 2019; Geng et al., 2022).

At the network and cellular level, the CNS is better understood thanks to a large body of histological and neuroanatomical data that has been acquired since the development of modern anatomy. Studies of central pathways have been spurred through microscopy, electrophysiology and - more recently - optogenetic tools for stimulating and reading out the function of neuronal circuits and neuron–glia interaction. In fact, over the past 30 years, neurobiology has largely benefitted from optical techniques - and, on the contrary, neuroscience has been a major driving force for optical techniques now collectively known as “neurophotonics” (Cho et al., 2016; Barros and Cunha, 2024).

At a macroscopic scale, our understanding of the brain has advanced significantly through the use of functional imaging techniques. Among these, functional magnetic resonance imaging (fMRI) is the most widely used both in research and clinical settings due to its non-invasive nature and high spatial resolution, which allows precise mapping of the entire brain. In the hospital, electroencephalogram (EEG) or positron emission tomography (PET) scans are often used. In addition, magnetic resonance imaging (MRI) techniques can also be used to image the CNS of murine models. However, these techniques will not be detailed in this review (for more information, see recent review Schwizer et al., 2006; Hanrahan et al., 2024).

Similar studies focusing on the ENS have lagged behind. Enteric circuits have mostly been studied using traditional histological or immunofluorescence protocols - techniques, which difficultly allow to apprehend the full complexity of ENS and to account for the diversity of different cell types and their interactions along the different intestinal segments. It was only recently that the use of highly resolved, and functional volume imaging techniques (Berghe et al., 2008; Schreiber et al., 2014a; Yissachar, 2020; Li et al., 2022 and see below) (either 3-D imaging of fixed, thick samples using confocal or light-sheet microscopies, or intravital, functional 2-photon imaging, …) have begun to provide new insights into this peculiar nervous system, its functions and interactions with other constituents of the intestine.

In this review, we will focus on how the development of 3-D imaging modalities has enabled an increasingly detailed knowledge of the structure and function of the ENS. This manuscript is organized as follows: (1), we will describe the traditional histological protocols that were developed to study the ENS with bright-field microscopy. (2), we then focus on the methodological developments that have enabled scientists to image the connections in between the different structures of the ENS in 2-D, before, (3), highlighting new optical imaging methods that reveal the 3-D organization of the ENS in its full complexity on fixed samples. Finally, (4) we will discuss the latest developments in *ex-vivo* and *in-vivo* optical imaging.

## 2 Images of a tubular network: from single ganglia to meshwork

**Figure 2:**
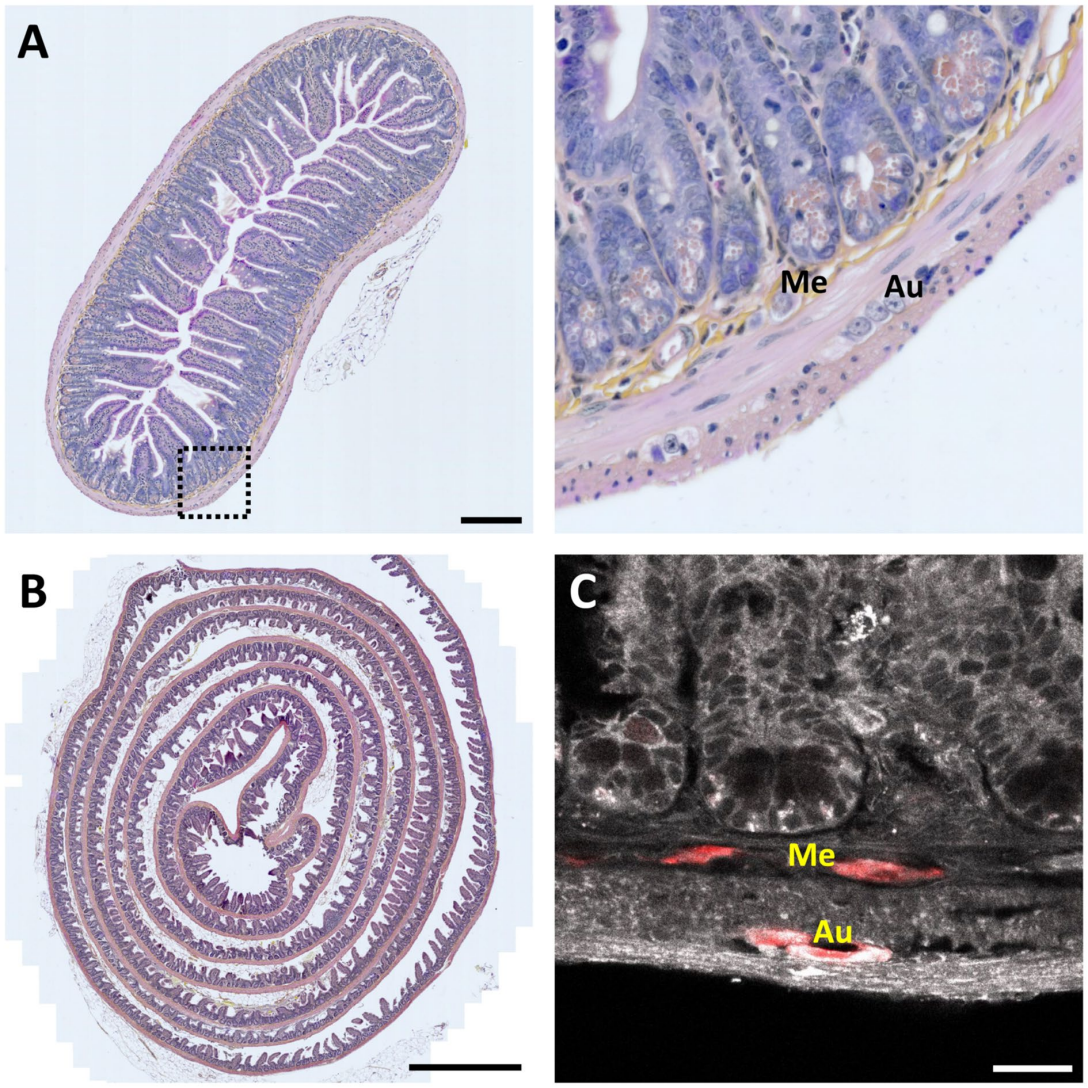
2-D histology of the intestine. **(A)** *Left*, 7-μm thin transversal section of mouse small intestine stained with Hematoxylin Eosin Saffron (HES). Scale bar 200 μm. *Right*, magnification view of the region indicated by a dotted square; Me – Meissner’s (submucosal) plexus; Au – Auerbach’s (myenteric) plexus (in order to find both plexuses in the same field of view, it was necessary to analyze several slices over more than an hour). **(B)** Mouse intestinal Swiss roll stained with HES. Scale bar, 1-mm. **(C)** Fluorescence image of a 7-μm thin mouse intestinal slice showing tissue autofluorescence (grayscale) and HuC /D neuronal immunolabeling (red). Scale bar: 25 μm.

In pathology, clinical neurogastroenterology and fundamental research, the ENS was mainly studied using classical histological techniques involving a sequence of steps (Figure 2):

- *Slicing.* After dissection and fixation, samples are embedded in paraffin or frozen to be cut into 5- to 7 -μm thin sections. In some cases, the sample was cut into numbered serial sections.
- *Staining.* These sections are then stained with conventional histological stains (e.g., Hematoxylin–Eosin (H&E), Nissl staining, …) or with immunohistochemistry techniques using specific antibodies against proteins marking specific cell types or a pathology-specific targets.
- *Imaging.* The sections are observed by bright-field microscopy for H&E, or by (confocal) fluorescence microscopy in the case of immunofluorescence (Figure 2C).
- *Rendering.* The consecutive 2-D images can then be aligned, dilated and digitally rendered to provide a (pseudo-) 3-D reconstruction.

This method allows visualizing the cell composition, single-cell morphology, and spatial organization of neurons and glial cells within the ENS (Figure 2A), and eventually observe pathological changes (Maia, 2000; Shayan et al., 2004). However, working on thin sections makes it challenging accurately reconstruct the complex 3-D ganglionated structure of the ENS.

While 3-D rendering provides a solution, it is a tedious and time-consuming process prone to errors. Issues such as navigation mistakes, tissue dilation or rupture, missing slices, misalignments, and discontinuities can introduce artifacts, complicating the reconstruction of larger neuronal or glial networks.

However, it is important to note that the 3-D morphology of the ENS is not well-adapted to be imaged on a single slice, and it also poses a problem already when looking at single slices. In fact, it is nearly impossible to analyze and understand such a structure from a single section. For example, while it is possible to observe single ganglia, finding both Auerbach’s and Meissner’s plexuses on a single section is anecdotic. To overcome these limitations, the “Swiss roll” technique was developed (Moolenbeek and Ruitenberg, 1981; Hazart et al., 2023; Dooley et al., 2024). In this approach, the intestine sample is incised longitudinally to expose the lumen, then rolled up on itself, with the luminal side facing inwards. The Swiss roll significantly increases the observable area and, therefore, the probability of finding both plexuses in the same field of view. However, it is still not possible to visualize the entire architecture, or to study the connections of the ENS with peripheral and central nerves, essential for a better understanding of this integrated and morphologically complex system (Figure 2B).

Finally, electronic microscopy can provide ultrastuctural images of the ENS, but often only small tissue volumes are explored (Delfino et al., 2024), however, for a bigger samples, block-face reconstruction was proposed (Nakanishi et al., 2020, 2023; Mantani et al., 2023). Thus, as we are more interested in multicellular assemblies and larger tissue volumes, electron microscopy is out of the scope of this review (see Zhang et al., 2024).

## 3 2-D whole-mount imaging of the ENS

**Figure 3:**
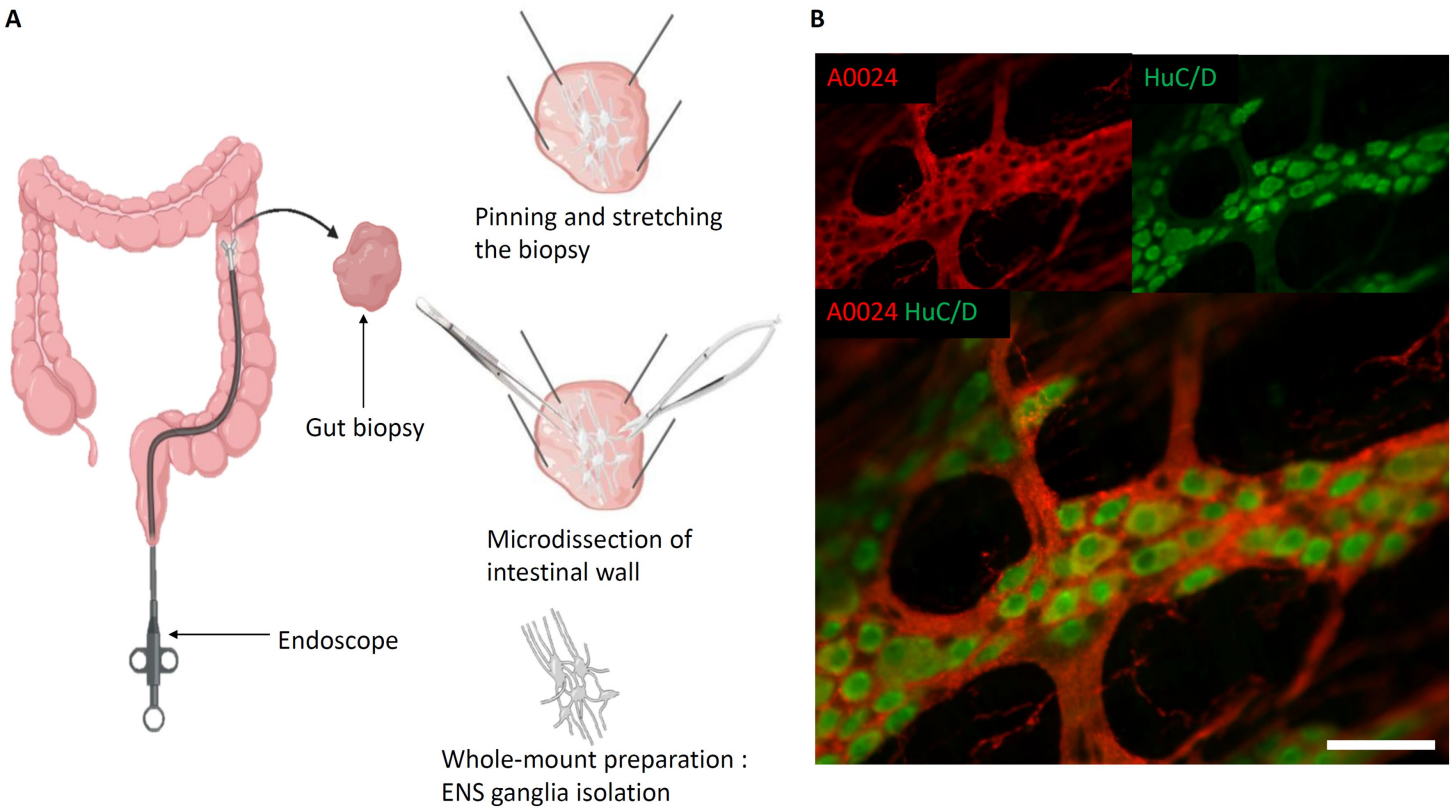
Whole-mount preparation of the Enteric Nervous System. **(A)** Steps of intestinal whole-mount preparation from biopsies (the same protocol can be applied on explants). *Left*, Biopsy performed with biopsy forceps. *Top right*, Biopsy pinned and stretched flat, submucosa oriented towards experimenter. *Middle right*, Separation of submucosa and mucosa layers. *Bottom right*, Isolated submucosa with submucosal plexus; **(B)** Myenteric whole-mount of mouse intestine. *Red,* A0024 pan tau immunofluorescence labeling; *green*, HuC/D neuronal immunofluorescent labeling. Scale bar: 50 μm. *(created with BioRender).*

As an alternative to slicing up the intestine into 2-D sections, the “whole-mount” technique (Lebouvier et al., 2010) was developed to study ENS ganglia and their connectivity (Figure 3A). It involves a careful dissection of the intestinal wall. For this, the intestine is opened longitudinally. Then, using micro-scissors and micro-forceps, the mucosa is gently removed to expose the submucosa. If a “whole-mount” preparation of the submucosal plexus is to be carried out, because of its inextricable links with certain elements of the submucosa, the latter is isolated from the muscularis and pinned and stretched on its support. On the other hand, to study the myenteric plexus, it is possible to isolate it from the 2 muscularis by micro-dissection. This technique, based on relatively difficult dissection, still does not preserve the tissular environment, yet, the technique can be applied to various species (Krammer et al., 1994; Nieves-Ríos et al., 2020; Sun et al., 2020; Mazzoni et al., 2021; Kigata et al., 2023; Gomez-Frittelli et al., 2024).

Also, opposed to sequential slicing, the whole-mount preparation preserves the ganglia of myenteric or submucosal plexuses, and thus permits to study their spatial relationship. In most studies, whole-mount preparations are immunolabeled and subsequently imaged using confocal laser scanning microscopy (CLSM) for the identification of different cell types and study their spatial arrangement (Teixeira et al., 1998; Boesmans et al., 2015b; Huang et al., 2021; Jooss et al., 2022) (Figure 3B).

It is also possible to work on living cells using this technique by carrying out the preparation in a medium that keeps the cells alive. In this context, it is possible to perform calcium imaging of ENS cells and record neuronal activity within the various ganglia (Gulbransen and Sharkey, 2009; Fried and Gulbransen, 2015; Cavin et al., 2023).

The whole-mount technique has also been used to study alterations in the ENS in IBD (Schneider et al., 2001; Neunlist et al., 2003; Talapka et al., 2014; Li et al., 2016), HSCR (Watanabe et al., 1999; Schäfer et al., 2003; Taniguchi et al., 2005), or PD (Pouclet et al., 2012; Phillips et al., 2013), by analyzing morphological alterations in nerve cells or quantifying variations in neuronal density and distribution in healthy and diseased conditions.

On the downside, the whole-mount method remains a relatively time-consuming and requiring technical skills. Furthermore, fluorescence imaging of whole-mount preparations generally requires the optimization of immunolabeling protocols due to the sample thickness. Finally, the extraction of surrounding connective tissue inevitably leads to a loss of context, altering the 3-D architecture. It is therefore impossible with this technique to study the enteric neuro-glial network in its natural habitat.

To better understand the ENS, it is therefore essential to study its 3-D architecture in intact tissue. To this end, many methods have been developed which involve imaging the ENS from thick tissues.

## 4 3-D imaging techniques for fixed intestinal tissue and the ENS

Different approaches are available for imaging the 3-D architecture of enteric neurons and glia in their native environment. These all require to image thick samples, which brings about new challenges both for introducing fluorescent labels and for microscopic imaging. Unlike the techniques presented earlier, these methods neither involve sectioning nor dissecting the sample. The integrity of the intestinal wall is thus maintained.

However, imaging tissue samples thicker than a couple of tens of μm inevitably faces the challenge of light scattering by the heterogeneous tissue composition. As classical wide-field and confocal microscopes rely on ‘ballistic’ (i.e., non-deviated) photons for image formation, they fail in a regime where many or even most photons are scattered. Deep tissue imaging hence requires either non-linear microscopy, typically 2-photon imaging or, (i), chemically modifying the sample to counteract the effect of light scattering (an approach known as “tissue clearing,” see for recent reviews Richardson and Lichtman, 2015; Neckel et al., 2016; Tainaka et al., 2016, 2018; Ariel, 2017; Tian et al., 2021), and, (ii), using microscopy techniques adapted to rapidly image large tissue volumes, typically confocal spinning disc (Gräf et al., 2005) or light-sheet microscopes (Elisa et al., 2018; Hillman et al., 2019; Poola et al., 2019).

Building on these advanced imaging strategies, recent studies have used sophisticated 3-D imaging techniques, tissue clearing and targeted labeling to refine our understanding of the ENS in both physiological and pathological contexts. These approaches have enabled unprecedented visualization of enteric neurons and glia while preserving tissue integrity, overcoming the limitations of traditional histological sectioning. By applying these methods to both animal models and human intestinal biopsies, researchers have significantly improved ENS mapping, revealing its intricate architecture and functional alterations in disease states such as cancer, inflammation, and developmental disorders. The following table (Table 1) summarize and compare the methodologies employed and the key findings across these studies.

**Table 1:** 3-D imaging of the ENS and their applications.

References	Approaches	Microscopy	Species	Innovation	Application	Key findings
Liu et al. (2011)	Immunolabeling Clearing (FocusClear©)	Confocal	Human	Improved imaging depth and signal-to-noise ratio for 3-D analysis of the human ENS.	Neuronal mapping	3-D reconstruction of nerve projections within the ENS. Facilitates structural and functional analysis of the myenteric and submucosal plexuses.
Liu et al. (2013)	Immunolabeling Clearing (FocusClear©)	Confocal	Human	First detailed 3-D reconstruction of the human enteric glial network. Morphometry of 3-D glial cell density, connectivity, and organization.	Glial mapping	First 3-D visualization of the human enteric glial network in the colonic mucosa. Identification of a complex and interconnected enteric glial network within the mucosa. Assessment of enteric glial cell number and distribution. Improved understanding of the role of enteric glia in normal and pathological intestinal functions.
Neckel et al. (2016)	Immunolabeling Clearing (PACT, CLARITY)	Confocal Light-sheet Transmission Electron	Human Mouse	Application of PACT tissue clearing for complex organs like the intestine Compatibility of tissue clearing with classical histology (H&E, Azan stain)	ENS mapping Pathological applications	Structural analysis of epithelial and vascular interfaces. Improved ultrastructural preservation using PACT at neutral pH. 3-D visualization of myenteric and submucosal plexuses. Integration of tissue clearing with traditional histology for pathology research.
Valès et al. (2019)	Immunolabeling Clearing (iDISCO) Cells culture	Light-sheet	Human Mouse	Primary cultures of human enteric glial cells to study their interaction with cancer stem cells. Murine xenograft models of human cancer stem cells to assess the impact of enteric glial cells on *in-vivo* tumorigenicity. Analysis of paracrine interactions between enteric glial cells and cancer stem cells using 3-D co-culture systems.	Colorectal cancer	Direct interaction between enteric glia and colon cancer stem cells. Enteric glia activates cancer stem cells and promotes tumor progression. Role of glial cells in colorectal tumorigenesis. Potential therapeutic targeting of this interaction for colorectal cancer treatment.
Graham et al. (2020)	Immunolabeling Clearing (Murray’s clear)	Confocal Light-sheet	Human	Development of a robust method for 3-D visualization of the human ENS without tissue sectioning using light-sheet fluorescence microscopy. Significant improvement over traditional histological techniques requiring tissue sectioning and laborious reconstruction.	Structural analysis of the ENS	New information on enteric neuronal morphology and connectivity. Potential method to study gastrointestinal diseases involving the ENS. Easier structural and functional analysis of the ENS.
Wang et al. (2021)	Adeno-Associated Virus (AAV) labeling Clearing (unknown)	Confocal	Mouse	Viral labeling and tracing of neuronal circuits of the enteric plexuses.	Functional mapping of the enteric plexuses	Identification of neuronal structures in the proximal enteric plexuses. Tool for studying neuroplasticity and digestive diseases in animal models.
Sun et al. (2021)	Immunolabeling Clearing (iDISCOace)	Light-sheet	Human Non-human primate Mouse		Intestinal inflammation	Sarm1-mediated neurodegeneration in the ENS reduces colonic inflammation. Neurodegeneration as a strategy to treat IBD (Crohn’s disease, ulcerative colitis).
Yuan et al. (2021)	Immunolabeling Clearing (CLARITY)	Confocal	Human	Detailed 3-D imaging and reconstruction of the human colon. Specific antibody targeting of human choline acetyltransferase (hpChAT), enabling mapping of cholinergic innervation.	Intestinal neurotransmission	Novel revelation of intrinsic cholinergic innervation of the human colon. Identification of cholinergic structures potentially involved in transit regulation and functional disorders like irritable bowel syndrome.
El-Nachef et al. (2022)	Transgenic fish model, Immunolabeling Clearing (RIMS)	Light-sheet	Zebrafish	Panoptic imaging and morphometry of all enteric neurons in the adult zebrafish. Whole-organ integrity, avoiding the need for tissue sectioning.	Neuronal quantification	Visualization of the enteric neuronal network in a model organism. Study of ENS development, regeneration, and digestive disorders associated with neuronal abnormalities.
Yuan et al. (2023)	Immunolabeling Clearing (CLARITY)	Confocal	Pig		Drug effects on the ENS	Loperamide reduces cholinergic innervation in the porcine colon upon repeated administration. Slowed intestinal transit affects neuronal plasticity. May explain certain side effects associated with prolonged loperamide use.
Eisenberg et al. (2024)	Immunolabeling Clearing (BABB)	Confocal	Human		Developmental disorders	New characterization of the ENS in the pediatric colon affected by HSCR. Identification of modifications in neuronal connectivity and distribution. Improved diagnosis and therapeutic approaches for this developmental disorder.

Advances in imaging and neuronal labeling have largely transformed the study of the ENS, revolutionizing our understanding by providing more comprehensive imaging while preserving tissue integrity and allowing for more in-depth functional analysis. The studies by Liu et al. (2011, 2013) and Graham et al. (2020) have improved visualization of glial and neuronal networks, resulting in deeper and more detailed imaging. Wang et al. (2021) introduced multicolor viral labeling, enabling the precise tracing of the enteric plexus, while El-Nachef et al. (2022) and Yuan et al. (2021) proposed advanced methods for quantifying and mapping neuronal networks.

At the same time, these imaging techniques have led to uncover the ENS’s involvement in various pathology (Valès et al., 2019) established for the first time a link between enteric glial cells and cancer, opening new perspectives in digestive oncology. Sun et al. (2021) highlighted a protective role of neurodegeneration against intestinal inflammation, a discovery that could reshape our understanding of IBD. Yuan et al. (2023) revealed an overlooked effect of intestinal transit-slowing drugs on the ENS, raising questions about the long-term administration of certain gastrointestinal treatments. Finally, Eisenberg et al. (2024) applied 3-D imaging to better characterizing pediatric colon abnormalities in HSCR.

Together, these studies refine our understanding of the roles of the ENS—going beyond its motor and nutritional functions—and demonstrate its involvement in cancer, inflammation, drug effects, and developmental disorders, providing new diagnostic tools and therapeutic targets.

## 5 Label-free 3-D tissue imaging

One difficulty when imaging large tissue volumes is antibody penetration. An alternative are transgenic mouse models in which fluorescent proteins are directed to specific cell types using genetic targeting. Yet another strategy is label-free imaging, which is of particular interest for (bio-) medical applications, too.

Autofluorescence (AF) detection has already been used for imaging various parts of the intestine (Castro-Olvera et al., 2024) upon mono- (Bhattacharjee et al., 2018) and multi-photon excitation (Rogart et al., 2008; Orzekowsky-Schroeder et al., 2011; Ricard et al., 2012; Ogawa et al., 2021) or using dynamic full-field optical coherence tomography imaging (D-FFOCT) (Durand et al., 2023) in healthy conditions but also for studying pathologies like HSCR (Aggarwal et al., 2013).

**Figure 4:**
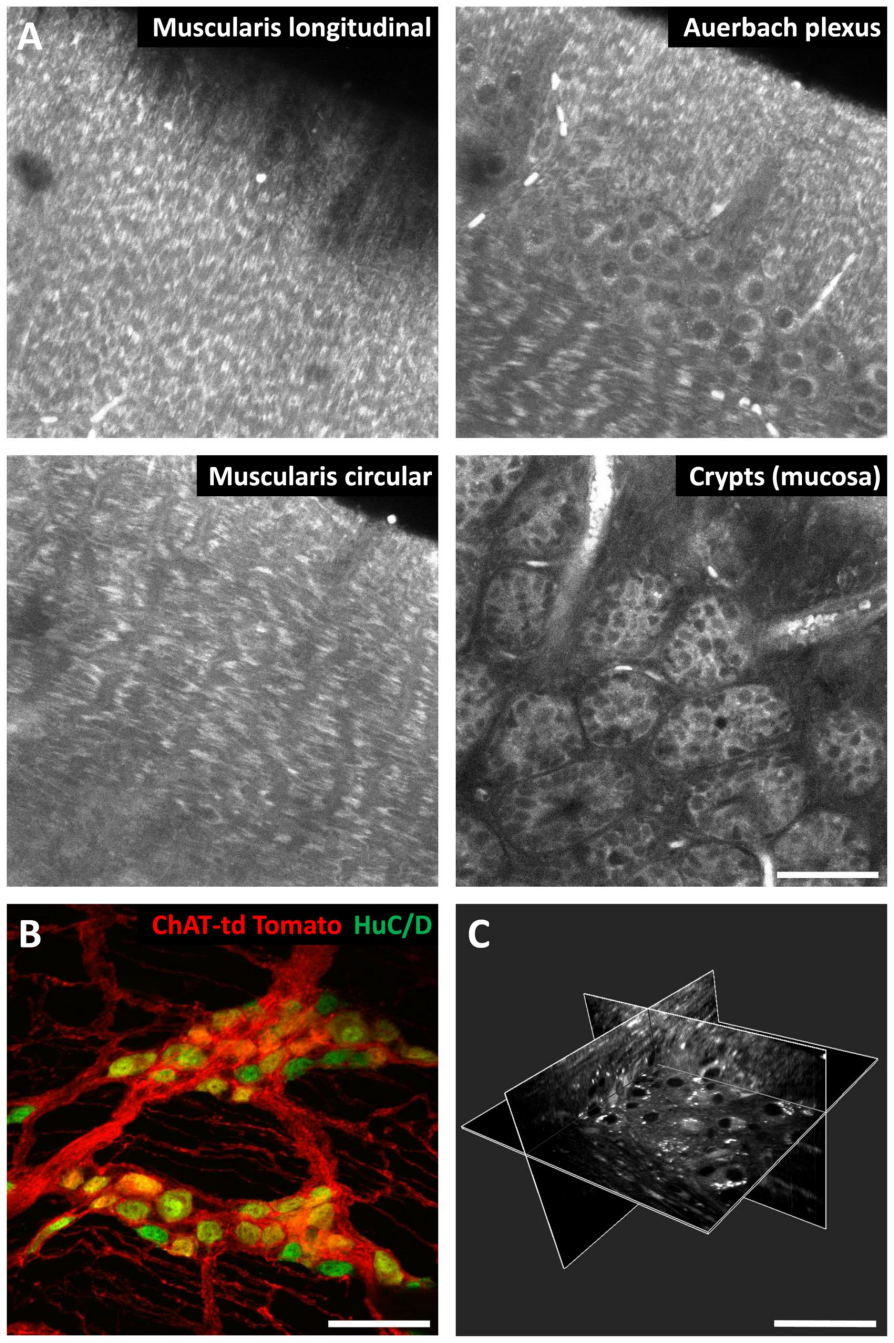
3-D autofluorescence imaging of the mouse intestine. **(A)** Autofluorescence images of a mouse small intestine sample at different depths. **(B)** Maximum-intensity projection over 34 μm of a *z*-stack of the small intestine wall of a choline acetyltransferase (ChAT) tdTomato mouse. *Red,* cholinergic neurons; *green*, HuC/D neuronal nuclei immunofluorescent labeling. **(C)** 3-D reconstruction from autofluorescence images of a mouse small intestine wall highlighting the Auerbach’s plexus. The white dots are most likely lipofuscin. Images were taken on a ZEISS LSM710 scanning confocal microscope **(A,B)** and on a custom 2-photon spinning-disk microscope (Hazart et al., 2023) **(C)**. Optical clearing was performed with UbiClear. Scale bar: 50 μm.

We recently developed a fast, large-area, 3-D AF and second-harmonic generation (SHG) imaging technique (Hazart et al., 2023) using a custom 2-photon spinning-disk microscope (Rakotoson et al., 2019) that combines the high spatial resolution and depth penetration of a 2-photon scanning microscope with the large field-of-view and speed of a light-sheet microscope. In our workflow, the intestinal sample is firstly submitted to a rapid (effective in a few minutes), non-toxic tissue clearing protocol (UbiClear©, Delhomme and Oheim, Patent WO2024089224A1 deposited 2023-10-26). Subsequently, 2-photon imaging of the rapidly cleared sample allowed us imaging across the entire mouse intestinal wall, from the serosa to the crypts, and identifying the various components of the ENS, such as the myenteric plexus and its neuronal cell bodies, or, in the submucosa, the submucosal plexus cells. Likewise, AF imaging allowed us tracing the connections between the various plexuses and other cells such as enteric glia. Of note, our technique can be also be combined with transgenic or immunofluorescence labels and thus can provide contextual images to specific (immuno-) labels (Hazart et al., 2023) (Figure 4).

Although AF is mainly detected under 2-photon microscopy, it is also possible to study AF under CLSM with excitation at 405 nm and 488 nm and we have recently shown that this technique, in addition to the mouse intestine (Hazart et al., 2023), can also provide detailed 3-D images of human colonic biopsies.

## 6 *Ex-vivo* imaging of enteric neuronal and glial activity

Imaging fixed tissue provides valuable insights into the organization of the ENS at the cellular and sub-cellular scale as well as its pathological malformations. Yet, it is not possible to obtain functional information about the ENS or to study the roles of individual cell types in the network.

To overcome this shortcoming, more and more laboratories employ transgenic mouse models expressing genetically encoded calcium (Ca^2+^) indicators (GECIs) combined with confocal or 2-photon Ca^2+^ imaging (Boesmans et al., 2015a, 2018; Fung and Vanden Berghe, 2020).

Depending on the cellular (or even sub-cellular) targeting strategy, Ca^2+^ imaging with GECIs is essential for understanding how identified population of neurons and glial cells interact to coordinate gastrointestinal functions. Some of these studies are based on the use of a device that permits an *ex-vivo* observation after intestinal resection to understand cellular mechanisms, such as mitochondrial Ca^2+^ uptake (Berghe et al., 2002), the activation of sensory neurons in the spontaneous colonic migratory motor complex (CMMC) (Hennig et al., 2015), or the modulation of neuronal activity (Bayguinov et al., 2010; Swaminathan et al., 2019; Li et al., 2022). In addition, studies focusing on glial cells have shown their role in the regulation of gastrointestinal homeostasis (Boesmans et al., 2013, 2015b, 2019).

A more recent *ex-vivo* calcium imaging technique using voltage-sensitive dyes has also been used on the ENS (Elfers et al., 2025). This technique simultaneously records changes in membrane potential in real time, providing direct information on neuronal excitability and synaptic transmission using voltage-sensitive dyes, and fluctuations in intracellular calcium using the usual calcium indicators.

Different *ex-vivo* preparations have been published to study intestinal motility. For this, after intestine resection, an elaborate perfusion device, in an organ bath, has been used, along with fine dissection techniques. This device has made it possible to demonstrate the existence of different repeated patterns of intestinal contraction. It has also been employed in pharmacological experiments to evaluate how certain drugs influence intestinal motility (Schreiber et al., 2014a, 2014b).

Similar *ex-vivo* devices maintaining stretches of the intestine alive for a limited time have been used in multimodal neuro-immune-microbiota crosstalk studies that enabled to uncover the close links of the ENS response to changes in the microbiota, whether or not related to an immune reaction, as in IBD (Yissachar et al., 2017; Yissachar, 2020; Gagliardi et al., 2021; Naim et al., 2024).

## 7 *In-vivo* imaging of enteric neuronal and glial activity

Although the above studies on explants have provided interesting new insights into activity motility patterns and roles of the microbiota, longitudinal or long-term functional studies require more intact preparations. In this context, *in-vivo* imaging techniques, similar to those used recently for the CNS, can provide relevant functional data. These include 2-photon imaging studies on intestinal segments after laparotomy (Goto et al., 2013; Kolesnikov et al., 2015), the use of an abdominal optical window (Rakhilin et al., 2016; Jiang et al., 2024), as well *in-vivo* Ca^2+^ imaging approaches (Rakhilin et al., 2016; Motegi et al., 2020; Nishimura et al., 2020; Jiang et al., 2024) and confocal laser endomicroscopy (Samarasena et al., 2016; Shimojima et al., 2020; Harada et al., 2021).

Like in other areas of neuroscience, the use of *in-vivo* 2-photon and 3-photon imaging has proven necessary for the detailed observation of small, subcellular structures that are not located near the surface of the preparation (Choi et al., 2018). The non-linear excitation confinement together with the efficient use even of scattered fluorescence (Oheim et al., 2001) allows routine imaging several hundred micrometers deep in intact intestinal tissue.

*In-vivo* imaging of more intact preparations goes along with often complex and invasive surgical protocols. Usually, a laparotomy is performed to expose part of the intestine outside the abdominal cavity for 2-photon imaging. Laparotomy derives from the Greek words *lapara*, meaning flank, and *tomy*, meaning incision. In surgical practice, this translates to a big incision in the abdomen to gain access to the peritoneal cavity. Usually, a standard laparotomy is an incision made in the midline along the *linea alba*. This technique revealed, for example the ability of ENS tissue to rebuild after injury (Goto et al., 2013) but also allowed investigating the immune response in the intestinal mucosa and the interactions between different types of epithelial cells and haematopoietic cells (Kolesnikov et al., 2015).

Another approach involves implanting an abdominal optical window for long-term *in-vivo* imaging (Rakhilin et al., 2016; Jiang et al., 2024). However, the first abdominal windows had the disadvantage of not holding the intestine in place. As a result, with gravity, the intestine very often adhered to the coverslip, leading to intestinal obstruction or chronic inflammation followed by the death of the individual. In addition, as the abdominal area was subject to mechanical stress and directly accessible to the individual (e.g., during grooming), leading to the detachment or rupture of the coverslip. Finally, peristaltic movements (sequential contraction of the muscles of the digestive tract allowing the food bolus to progress), heartbeats and respiratory movements can compromise the ability to perform high-resolution imaging of the ENS, although such movement artefacts can, in part, be counteracted by motion-triggered acquisitions.

In response to these challenges, some laboratories have improved the abdominal optical window device, creating devices with 3-D elements (‘clips’) that hold the intestine in place in the abdominal cavity and stabilize it in an attempt to reduce physiological movements of the intestine. This not only reduces animal mortality, but also allows for long-term monitoring. Combined with 2-photon imaging or *in-vivo* confocal imaging using long working distance objectives, detailed 4-D (3-D + time) imaging is then possible (Rakhilin et al., 2016; Jiang et al., 2024).

In addition, with either laparotomy and abdominal optical window combinations of 3-D morphological and *in-vivo* Ca^2+^ imaging are possible (Rakhilin et al., 2016; Motegi et al., 2020). Similarly, *in-vivo* Ca^2+^ imaging and EEG have been associated to gain a better understanding of the interactions between the vagus nerve and the ENS (the gut-brain axis) (Nishimura et al., 2020; Jiang et al., 2024).

Finally, confocal laser endomicroscopy is another *in-vivo* imaging approach that has been employed for both human and animal models. Similar to classical CLSM, it involves focusing a low-power laser beam and scanning an area point by point to create a 3-D image. The main difference is that the endoscope optics relays the imaging plane inside the intestine. The laser beam then excites exogenous fluorescent agents (e.g., NeuroTrace©, Cresyl Violet) administered either topically or systemically. This technique can therefore be used to visualize neuronal cells and nerve fibers of the ENS. However, compared with 2-photon imaging, the spatial resolution remains low, limited mainly by the typically lower numerical aperture and by peristaltic movements. Nevertheless, microendoscopy is effective for diagnosing pathologies, such as Hirschsprung’s disease, which is characterized by a near-total absence of enteric neurons in the terminal part of the colon (Samarasena et al., 2016; Shimojima et al., 2020; Harada et al., 2021).

Taken together, a growing body of ENS *in-vivo* imaging studies starts to unravel the details of intestinal physiology.

*In-vivo* imaging currently is the only way to study the function and interactions of different cell types in the intestine. However, experimental difficulties, ethical issues and the often reduced spatial resolution due to movement artefacts make *in-vivo* imaging much less effective for than *in-vitro* studies for detailed 3-D morphological investigations of the ENS and for detecting potentially subtle, pathological alterations at cellular and sub-cellular resolution. Another important question is that of the relevance of the animal model. Do the predominantly used mice and accessible intestinal segments adequately describe the anatomy and physiology? Are there important differences to the ENS in humans? In the case of neurodegenerative diseases, which by definition evolve on the long term and are most often manifest only at an advanced age, the lifespan of the animal model might be too short for a “natural” development and observation of disease progression. Genetic or pathogen-induced animal models on the other hand replicate some, but not all forms of human disease. What is more, the animal models used for understanding these diseases evolve in highly controlled environments (food, cage, limited social interactions, often poor environment), far removed from the environmental challenges and lifestyle of human beings. Yet the development of diseases, like PD or autism, is multifactorial and animal models remain a poor proxy for the human etiology.

While animal tissues are more easily available, their relevance to human diseases is limited by physiological differences, as showcased above. Furthermore, obtaining human tissue samples is highly regulated, constrained by numerous ethical and legal rules. Post-mortem samples or biopsies provide invaluable insights into human-specific pathology but may lack temporal resolution for studying disease progression. For this reason, techniques using induced pluripotent stem cells (iPSCs) and organoids derived from human cells have been developed. However, these approaches remain technically demanding and costly. Animal models, though imperfect, offer practical advantages in terms of availability, experimental manipulation, and the ability to observe long-term effects within a living organism. These differences highlight the complementary roles of human tissues and animal models in understanding human pathologies.

## 8 Conclusion

To better understand the ENS, its fine morphology and detailed functions, it remains essential to have access to a large panel of preparations and techniques. At present, only a combination of approaches allows studying the ENS at the different temporal and spatial scales relevant for deciphering its functions. Also, it remains inevitable to use different observables (molecular, histological, functional). Each of these methods provides specific information, helping us to better appreciated the complexity of the ENS and its role in health and disease.

We have seen that traditional histological techniques provided crucial information on the structure and organization of the ENS. However, 2-D observations are unable to capture the complex morphology of this tubular, multi-layer nervous system and give limited insight into cellular subtypes and connectivity.

With the “whole-mount” technique, communication between ganglia is observable, but the study of the ENS remains confined to distorted and context-free tissue in 2-D, significantly limiting our understanding of this particularly complex and integrated system. The development of 3-D imaging techniques has finally enabled high-resolution studies of the structure of the ENS and its complex network, including the communication between the 2 plexuses. Nevertheless, for a full understanding the physiology of the ENS and studying interactions on the intestine-brain axis, *in-vivo* imaging remains not the most effective but also the only technique for the moment.

## Glossary

3-D: Three-dimension
AAV: Adeno associated virus
AD: Alzheimer’s disease
AF: Autofluorescence
Au: Auerbach’s plexus
Ca^2+^: Calcium
ChAT: Choline acetyltransferase
CLSM: Confocal laser scanning microscopy
CMMC: Colonic migratory motor complex
CNS: Central nervous system
D-FFOCT: Dynamic full-field optical coherence tomography
EEG: Electroencephalogram
ENS: Enteric nervous system
fMRI: Functional magnetic resonance imaging
GECI: Genetically encoded calcium indicator
H&E: Hematoxylin–Eosin
HES: Hematoxylin–Eosin-Saffron
HSCR: Hirschsprung’s disease
IBD: Inflammatory bowel disease
iPSC: Induced pluripotent stem cell
Me: Meissner’s plexus
MRI: Magnetic resonance imaging
PD: Parkinson’s disease
PET: Positron emission tomography
SHG: Second-harmonic generation
